# Reliable quantification of BOLD fMRI cerebrovascular reactivity despite poor breath-hold performance^☆^

**DOI:** 10.1016/j.neuroimage.2013.07.007

**Published:** 2013-12

**Authors:** Molly G. Bright, Kevin Murphy

**Affiliations:** Cardiff University Brain Research Imaging Centre (CUBRIC), School of Psychology, Cardiff University, CF10 3AT Cardiff, UK

**Keywords:** Cerebrovascular reactivity, Breath-hold, BOLD, Patients, Repeatability

## Abstract

Cerebrovascular reactivity (CVR) can be mapped using BOLD fMRI to provide a clinical insight into vascular health that can be used to diagnose cerebrovascular disease. Breath-holds are a readily accessible method for producing the required arterial CO_2_ increases but their implementation into clinical studies is limited by concerns that patients will demonstrate highly variable performance of breath-hold challenges. This study assesses the repeatability of CVR measurements despite poor task performance, to determine if and how robust results could be achieved with breath-holds in patients. Twelve healthy volunteers were scanned at 3T. Six functional scans were acquired, each consisting of 6 breath-hold challenges (10, 15, or 20 s duration) interleaved with periods of paced breathing. These scans simulated the varying breath-hold consistency and ability levels that may occur in patient data. Uniform ramps, time-scaled ramps, and end-tidal CO_2_ data were used as regressors in a general linear model in order to measure CVR at the grey matter, regional, and voxelwise level. The intraclass correlation coefficient (ICC) quantified the repeatability of the CVR measurement for each breath-hold regressor type and scale of interest across the variable task performances. The ramp regressors did not fully account for variability in breath-hold performance and did not achieve acceptable repeatability (ICC < 0.4) in several regions analysed. In contrast, the end-tidal CO_2_ regressors resulted in “excellent” repeatability (ICC = 0.82) in the average grey matter data, and resulted in acceptable repeatability in all smaller regions tested (ICC > 0.4). Further analysis of intra-subject CVR variability across the brain (ICC_spatial_ and voxelwise correlation) supported the use of end-tidal CO_2_ data to extract robust whole-brain CVR maps, despite variability in breath-hold performance. We conclude that the incorporation of end-tidal CO_2_ monitoring into scanning enables robust, repeatable measurement of CVR that makes breath-hold challenges suitable for routine clinical practice.

## Highlights

• Cerebrovascular reactivity can be measured despite poor breath-hold performance.
• Measuring end-tidal CO_2_ accounts for varying breath-hold ability and consistency.
• Breath-holds can provide CVR maps with “excellent” repeatability.
• Breath-holding is a robust tool for assessing vascular health in patient studies.

## Introduction

Cerebral blood vessels are constantly constricting and dilating to regulate and stabilise the supply of blood to downstream brain tissue. When functioning properly, these mechanisms protect against fluctuations in blood pressure (i.e., cerebral autoregulation) and support increases in metabolism associated with neural activation. However, when these vascular mechanisms are disrupted, such as in the presence of arterial plaques or vessel hardening, the downstream tissue may be at risk.

Cerebrovascular reactivity (CVR) is the response of cerebral blood vessels to vasoactive stimuli such as exogenous drugs (e.g., acetazolamide) or arterial CO_2_ levels. The potent vasodilatory nature of arterial CO_2_ in humans, described by Kety and Schmidt (1948), can be manipulated using inhalation of air with increased CO_2_ content to induce hypercapnia. Voluntary respiratory challenges (e.g., breath-holding or deep breathing) can also cause more transient changes (Bright et al., 2010). CVR measurements can assist in the diagnosis of cerebrovascular disease and aid treatment planning in these patients. For example, a recent meta-analysis of the CVR literature revealed strong association between CVR impairment and the risk of ischaemic event in stenosis patients (Gupta et al., 2012).

Blood Oxygenation Level Dependent (BOLD) contrast functional magnetic resonance imaging (fMRI) takes advantage of the differing magnetic properties of oxygenated and deoxygenated haemoglobin to noninvasively measure changes in local vascular properties (Bandettini et al., 1992; Kwong et al., 1992; Ogawa et al., 1992). Combining this imaging modality with vasoactive stimuli, such as manipulations of arterial gas content, is a robust means for characterising CVR throughout the brain (Shiino et al., 2003). Currently, clinical CVR studies involving elevated CO_2_ levels are typically achieved using gas administration during scanning (Heyn et al., 2010), but this may not be appropriate or feasible for all research and patient scenarios.

Breath-holds offer a simple alternative method for elevating arterial CO_2_ levels, and the BOLD CVR measurements achieved using these respiratory challenges are similar to those obtained using inhaled CO_2_ methods (Kastrup et al., 2001). The relative simplicity of a breath-hold experiment has many advantages in clinical studies: it removes the uncomfortable presence of a face mask within the head coil, and by being self-directed it allows the patient to abort the task at any point, improving real-time safety and reassurance for the patient. Several other clinical areas such as cardiac imaging (Mahnken et al., 2007) already incorporate breath-holds, demonstrating that it is a well-tolerated technique.

However, the specifics of breath-hold execution determine the magnitude and dynamics of the BOLD signal response, and may impact how CVR is quantified. Firstly, the issue of breath-hold duration is potentially critical: although even short breath-holds (3 s) can cause a measurable BOLD response, the number of voxels exhibiting significant BOLD signal changes is increased with the duration of the breath-hold (Abbott et al., 2005). Longer breath-holds also offer more robust and repeatable BOLD responses across scanning sessions spanning weeks (Magon et al., 2009). Thus, although a 20-second breath-hold may be desirable to achieve a repeatable, robust BOLD response, this challenge may be too difficult for certain patient groups.

Several studies have established good practice guidelines for repeatable breath-hold fMRI experiments in healthy subjects. For example, paced breathing between challenges provides a more consistent baseline condition, from which the breath-hold signal response can be more accurately extracted, exhibiting less inter-trial variability (Scouten and Schwarzbauer, 2008). In addition, breath-holds after expiration may offer better reproducibility: following normal exhalation, the lungs return to the state of “functional reserve capacity,” at which the lungs, chest, and diaphragm are resting at equilibrium. This is a more repeatable starting point for breath-hold challenges, compared with self-directed inspirations that do not involve a natural equilibrium position (Scouten and Schwarzbauer, 2008). End-expiration breath-holds also avoid the biphasic BOLD signal response that is observed in end-inspiration challenges (Thomason and Glover, 2008).

However, most of these studies have not addressed the question of whether breath-hold challenges are useful for studying CVR in clinical patients. A recent publication examining the feasibility of mapping BOLD CVR in patients showed successful results in 364 of 434 patients using gas administration techniques; the authors specifically state that while breath-holds may provide similar information, “unfortunately, the degree of cooperation for breath holding will vary too much over the range of patients with neurologic diseases to be considered routinely useful” (Spano et al., 2013). Indeed, it may be more difficult for patients to execute a breath-hold, even those considered “short” by research standards (e.g., 15 s). There is likely to be reduced time to familiarise patients with the breath-hold technique prior to scanning when in a clinical environment. Breath-hold performance may therefore be more variable, and less repeatable, in patient studies.

In this study, we simulate patient behaviour in healthy volunteers. This allows more flexibility in our experiment duration and removes the confounds of age-related or pathological brain changes in order to isolate the effects of poor breath-hold performance on BOLD CVR measurement. Based on previous findings in healthy volunteers (Murphy et al., 2011), we hypothesise that using end-tidal gas measurements will enable CVR to be mapped in a robust, repeatable manner, across the brain, despite the underlying patient “misbehaviour.” End-tidal CO_2_ values, which represent the averaging of alveolar values, are linked to arterial CO_2_ levels; fluctuations in end-tidal CO_2_ are commonly used as a surrogate for fluctuations in arterial CO_2_ (Robbins et al., 1990). The change in arterial CO_2_ levels associated with the breath-hold challenge is the direct cause of the vascular response (and BOLD signal change), and thus measuring these CO_2_ effects should provide a full model of the hypercapnia stimulus regardless of how the challenge was actually executed. Inter-subject differences in the physiological response to the breath-hold challenge are also fully incorporated into the CO_2_ data collected. The results of this study allow us to offer advice on how to achieve reproducible CVR measurements in patients using BOLD fMRI and breath-hold challenges.

## Methods

### Data acquisition

Twelve healthy subjects (aged 32 ± 6 years, 5 female) were scanned using a 3T GE HDx scanner (Milwaukee, WI, USA) equipped with an 8-channel receive head coil. Functional data were acquired using a BOLD-weighted gradient-echo echo-planar imaging sequence (TR/TE = 2000/35 ms; FOV = 22.4 cm; 35 slices, slice thickness = 4 mm; resolution = 3.5 × 3.5 × 4.0 mm^3^). Six breath-hold scans and one resting scan were collected in a randomised order, each consisting of 165 volume acquisitions (5.5 min). A whole-brain high-resolution T1-weighted structural image was acquired (resolution = 1 × 1 × 1 mm^3^), for the purposes of image registration. Cardiac pulsations were recorded using a finger plethysmograph and respiration was measured via respiratory bellows. Expired gas content was continuously monitored via a nasal cannula, and O_2_ and CO_2_ data were recorded (AEI Technologies, PA, USA). This study was approved by the Cardiff University School of Psychology Ethics Committee, and all volunteers gave written informed consent.

#### Breath-hold paradigms

**Fig. 1:**
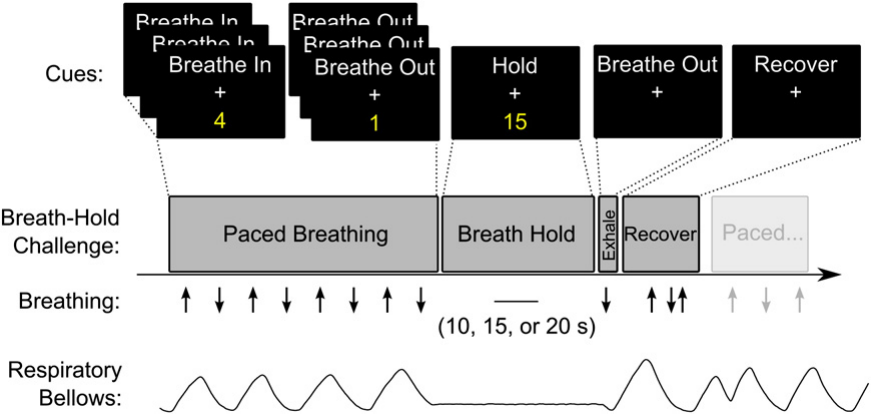
Schematic of the breath-hold paradigms. Each challenge was preceded by paced breathing (6 s period) ending on an exhalation, and at the end of the breath-hold a small exhalation was made to provide accurate end-tidal measurements. Following free “recovery” breathing, the paced breathing was resumed. An example trace from the respiratory bellows is provided.

The breath-hold paradigms were cued using textual instructions viewed through a mirror positioned on the head coil. The challenge is schematically presented in Fig. 1. In the periods between breath-hold challenges, subjects followed paced breathing commands at a rate of 1/6 Hz. A countdown timer indicated the number of seconds remaining before the next challenge. The breath-hold always followed an expiratory breath. A new countdown, beginning at either 10, 15 or 20 s, indicated the time remaining in the challenge. Upon completion, the subject was cued to “Breathe Out” to expel any remaining gas and provide an accurate end-tidal CO_2_ measure, before having several seconds of un-paced recovery breathing. Paced breathing was then resumed.

We considered two performance factors when simulating patient breath-hold data: ability and consistency. Patient ability reflects a physical limit during breath-holding: certain individuals or patient groups may not be able to complete the target breath-hold length and must abort the challenge prematurely. Patient consistency reflects the potential for variable performance across multiple breath-hold trials.

All breath-hold scans consisted of 6 breath-hold challenges, cued in the manner described above. In the *All10*, *All15* and *All20* breath-hold runs, the length of the breath-hold was held constant throughout the run at 10 s, 15 s and 20 s, respectively. In the *Avg12.5*, *Avg15* and *Avg17.5* runs, a combination of the three breath-hold lengths was chosen so that the average breath-hold length was 12.5 s, 15 s and 17.5 s, respectively. These scans simulate different breath-hold consistency levels at low, medium, and high ability levels.

The start times of the 6 breath-hold challenges were held constant (equally spaced) in all of the 6 paradigms, and were always preceded by 24 s of paced breathing. When breath-holds were shorter than 20 s, the “recovery” period was extended from the original 6 s up to 11 s or 16 s as necessary.

### Data analysis

**Fig. 2:**
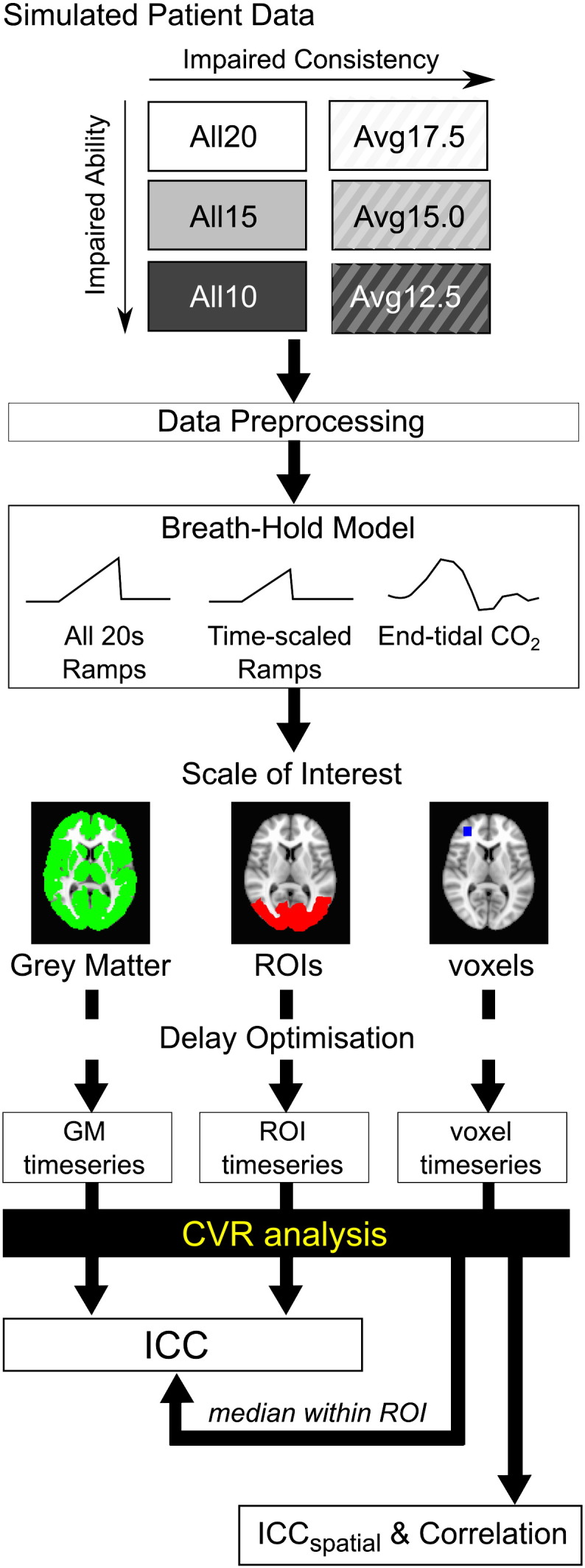
Schematic of analysis pipelines used in this study. Six breath-hold scans were acquired to simulate varying levels of ability and consistency. Following standard data preprocessing, CVR was calculated as follows: a breath-hold model was selected, a scale of interest defined, and an optimal delay time between breath-hold regressor and data determined prior to including the regressor in a general linear model. CVR results obtained using grey matter or ROI mean timeseries, as well as the median CVR value obtained from voxelwise values, were used to calculate the intraclass correlation coefficient (ICC) across the 12 subjects and 6 scans. The ICC quantifies the repeatability of a subject's CVR measure despite poor breath-hold performance. The voxelwise CVR values were also compared within an individual subject dataset using correlation analysis and ICC_spatial_; both of these parameters assess the consistency of spatial patterns of voxelwise CVR values within the grey matter of one subject.

A schematic of the analysis pipelines used in this study is presented in Fig. 2.

#### Data preprocessing

The functional data were volume registered, motion corrected, time-shifted to a common temporal origin, brain extracted, and converted to percentage BOLD change (AFNI, http://afni.nimh.nih.gov/afni (Cox, 1996)). A grey matter (GM) segmentation map in functional image space was calculated from the mean volume of the resting dataset using a T1-mapping technique (Bodurka et al., 2007). All other regions of interest were defined using the MNI atlas (Mazziotta et al., 2001). The functional data were registered to the high-resolution structural image, which was then normalised to the MNI152 standard brain (MNI152, nonlinearly derived, McConnell Brain Imaging Centre, Montreal Neurological Institute, McGill University, Montreal, Quebec, Canada). This transformation was inverted and applied to transform regions of interest (ROIs) from standard space into the functional space of each subject. These transformed regions were then masked by the subject-specific GM mask.

#### Breath-hold model

Three regressor categories were used to analyse the %BOLD signal change associated with the breath-hold challenges.

• *All 20s Ramps* — all breath-holds are modelled by a 20-second linear ramp, convolved with an HRF.
• *Time-scaled Ramps* — the duration of each breath-hold is known (10, 15, or 20 s) and challenges are modelled by a ramp lasting the correct length with an amplitude proportional to the duration and convolved with an HRF.
• *End-tidal CO_2_* — the P_ET_CO_2_ data were extracted and convolved with an HRF. These regressors were not normalised, but maintained in absolute units of mm Hg.

The *All 20s Ramps* pipeline presumes that all breath-holds last 20 s. This model, which does not incorporate any respiratory monitoring in the analysis, does not take into account variation in patient performance simulated in the six breath-hold scans. The BOLD breath-hold response has been shown to increase linearly with time (Murphy et al., 2011), and thus the challenges were modelled as linear ramps lasting 20 s with a final amplitude of 1. The *Time-scaled Ramps* pipeline assumes that any variance in the duration of breath-hold is known (i.e., from the respiratory bellows recordings), and challenges were modelled as a constant linear slope over the “corrected” period of time. Finally, the *End-tidal CO_2_* pipeline makes no assumption about breath-hold length, but instead models the challenge response with the recorded end-tidal CO_2_ values. In this study, we hypothesise that monitoring end-tidal CO_2_ is a more accurate representation of the subject's physiological response to a breath-hold than the duration of the breath-hold challenge, and thus a better fit to the BOLD response.

#### Scale of interest

Although measuring CVR in global grey matter provides insight into the overall responsiveness of the cerebrovasculature to the breath-hold hypercapnia stimulus, it is critical that our analysis is sensitive to regional variations in CVR within the brain in order to identify threatened or damaged tissue areas in clinical patients. This could be performed by averaging the BOLD signal within a region of interest, which has advantageous contrast- and signal-to-noise effects. If the noise properties of the data are sufficient, it may also be possible to map CVR for every voxel in the brain; if the CVR accuracy is not compromised, this method would provide the most insight for an observing clinician. To assess the limitations of measuring regional or local CVR, three “scales of interest” were considered.

• *Grey matter* (*GM*)
  The mean timeseries within the subject-specific GM mask was calculated.
• *Regions of interest* (*ROIs*)
  Regions were identified using the MNI atlas (Mazziotta et al., 2001) and transformed into the individual subject functional data space: frontal lobe, parietal lobe, temporal lobe, occipital lobe, insula, thalamus, and putamen. The transformed ROIs were then masked using the subject-specific GM mask. The mean timeseries within these ROIs were calculated.
• *Voxels*
  Each voxel timeseries was considered independently.

The timing of BOLD response to respiratory challenges varies across the healthy brain (Bright et al., 2009), and this temporal mismatch must be taken into account prior to further analysis. The breath-hold regressors were shifted with respect to the data to account for delays between the breath-hold challenge, the physiological response, and the resulting BOLD signal changes. This delay was optimised using cross correlation between the interpolated regressor and the appropriate timeseries (GM, ROI, or voxel), shifted in steps of 0.1 s.

#### Cerebrovascular reactivity analysis

Cerebrovascular reactivity was measured using the framework of a general linear model (GLM), where the beta-weight calculated for the breath-hold regressor reflects the %BOLD signal change associated with the hypercapnia challenges. When end-tidal CO_2_ regressors are used, this value is quantified in units of %BOLD/mm Hg. A 3-degree polynomial was also included to remove slow signal drift.

For each breath-hold scan, CVR was measured for the average timeseries from within GM and within each ROI. The median values of voxelwise CVR within the GM and ROI masks were calculated and compared to assess the impact of the scale of analysis on CVR results.

#### Repeatability assessment

To quantify the repeatability of our CVR measurements, we employed the intra-class correlation coefficient (ICC), which reflects the ratio between the data variance of interest (inter-subject CVR differences) and the total data variance, including differences between the six breath-hold scans. Under our assumption that the underlying vascular physiology for a given subject does not change during the scanning session, the ICC quantifies whether the CVR value measured for a given subject is repeatable across different breath-hold performances. Low ICC values (near 0) indicate that CVR measurements vary greatly across the six scans, relative to the variation between subjects. High ICC values (near 1) indicate there is minimal variance across the six scans, relative to inter-subject differences.

There are multiple forms of the ICC that are tailored to the specifics of a given experiment; we apply the ICC(2,1), which considers the six breath-hold scans to be a random sample of a larger population of possible breath-hold performances. This statistic therefore reflects a broader range of breath-hold ability and consistency levels than that which is simulated in the current study. The calculation of ICC(2,1) is described in detail in the literature (Shrout and Fleiss, 1979). The ICC values were also transformed into F-statistics to obtain the associated p-values.

Firstly, we use the ICC to test the repeatability of our calculation across the 12 subjects and 6 breath-hold scans using the *All 20s Ramps*, *Time-scaled Ramps*, and *End-tidal CO_2_* regressors. The CVR values measured using the GM or ROI timeseries, and the median voxelwise CVR values within the GM or ROI masks, were compared.

Secondly, we use ICC_spatial_ to measure whether the spatial distribution of voxelwise CVR for an individual subject is reproducible (Caceres et al., 2009; Raemaekers et al., 2007). This indicates whether breath-hold challenges robustly map CVR heterogeneity across the brain. The CVR values of individual voxels within a subject's GM mask were compared across the six breath-hold datasets. The ICC_spatial_ values for the three breath-hold models were compared across subjects.

This spatial distribution repeatability was also assessed between pairs of CVR maps: the *All15* and *Avg15* CVR maps (representing similar ability but different consistency levels) *All10* and *All20* CVR maps (representing different ability but similar consistency levels), and *Avg12.5* and *Avg17.5* CVR maps (representing difference in both performance factors) were compared by correlating the voxelwise CVR values within the GM mask. Outlier voxels were identified as having CVR values 3 standard deviations above the mean GM value or displaying negative CVR values (although physiologically possible, the interpretation of negative CVR values is outside the scope of this paper), and the subject-specific GM masks were adjusted such that any voxels considered an outlier in any dataset were removed from analysis. The correlation coefficients were transformed into z(r) scores using the Fisher r–z transform, and paired t-tests were used to determine significant differences (p < 0.05, Bonferroni corrected for multiple comparisons).

## Results

**Fig. 3:**
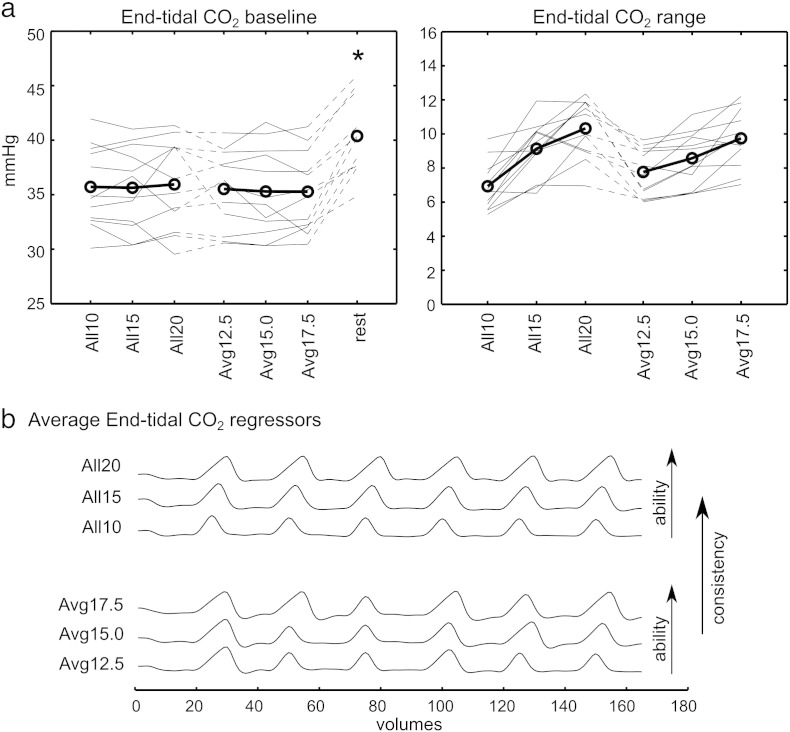
Summary of the end-tidal CO_2_ effects associated with the six breath-hold scans. a) The baseline and range of end-tidal CO_2_ values for each subject are shown and the group mean plotted (circles). The baseline end-tidal CO_2_ values were significantly lower in all six breath-hold scans compared to the resting scan (*p < 0.0005, paired t-tests, Bonferroni corrected for multiple comparisons). b) The group average end-tidal CO_2_ regressor for each of the six scans, accounting for temporal shifts between subjects.

All subjects completed the six functional breath-hold scans. A summary of the end-tidal CO_2_ effects related to the 6 breath-hold paradigms is shown in Fig. 3. The baseline end-tidal CO_2_ levels were significantly reduced in all six breath-hold scans relative to the resting scan. This hypocapnia is likely related to the paced breathing; subjects often breathe more deeply when consciously controlling their respiration rate. Also, as expected, the breath-hold duration (performance ability level) was coupled to the CO_2_ response: the end-tidal CO_2_ range increased as the average breath-hold duration throughout the scan was lengthened (e.g., from the *All10s* to the *All20s* datasets). Fig. 3 contains the end-tidal CO_2_ regressors for each of the 6 scans (group average, accounting for differing delay times across subjects).

### CVR repeatability

**Fig. 4:**
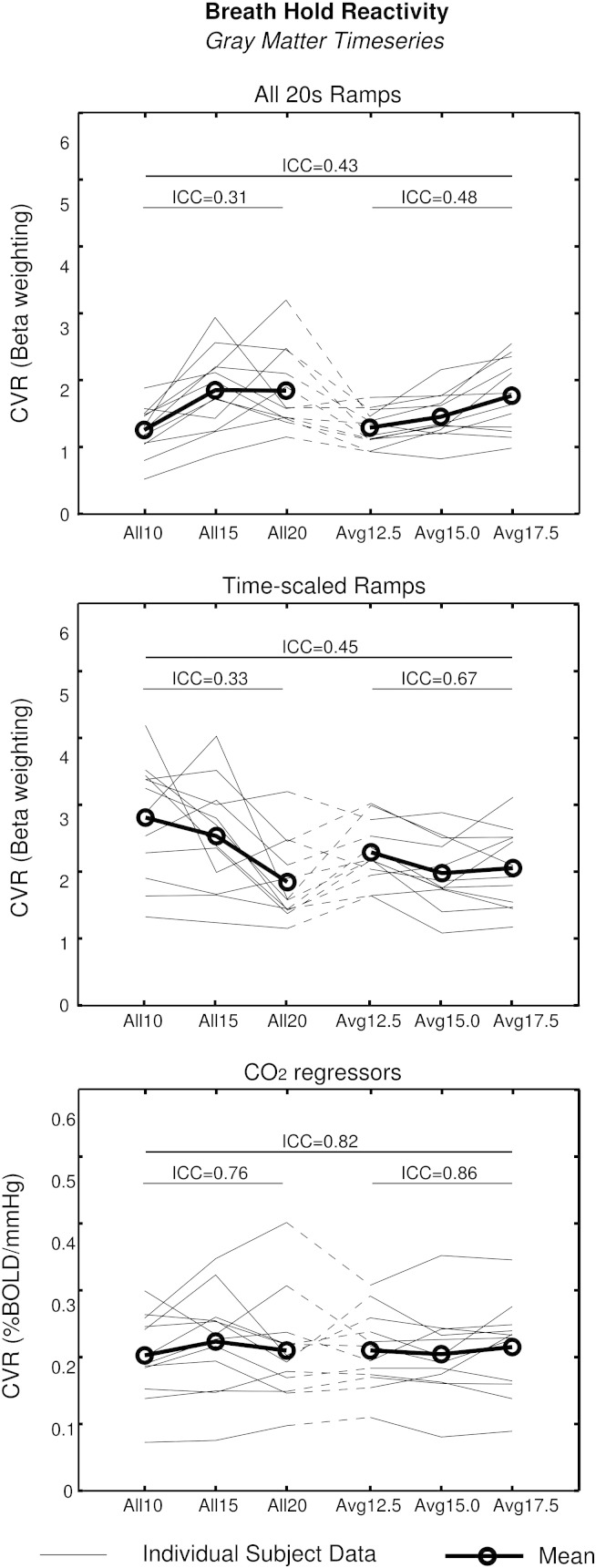
Repeatability of CVR values obtained from the GM average timeseries across the six breath-hold scans, for each of the 3 breath-hold models: *All 20s Ramps*, *Time-scaled Ramps*, and End-tidal *CO_2_* regressors. CVR values from individual subjects (thin lines) and the group average (thick lines, circles) are shown. The ICC values were calculated from all six breath-hold scans, the three consistent scans (*All10*, *All15* and *All20*) and the three inconsistent breath-hold scans (*Avg12.5*, *Avg15.0* and *Avg17.5*) separately, and the results are indicated on the graph.

The repeatability of the GM CVR values, obtained using the GM average timeseries, is illustrated in Fig. 4. The ICC values for the *All 20s Ramps*, *Time-scaled Ramps*, and *End-tidal CO_2_* analyses were 0.43, 0.45, and 0.82, respectively. All values were significant (p < 0.05). The ICC was recalculated using the three scans with consistent performance (*All10*, *All15*, *All20*) and the three with inconsistent performance (*Avg12.5*, *Avg15*, *Avg17.5*), and the results are also provided in Fig. 4. The correlation between the group mean CVR values and the group mean range in end-tidal CO_2_ values across the six scans was assessed to determine the presence of biases in the three breath-hold analysis methods. CVR was significantly correlated with the CO_2_ range in the *All 20s Ramps* analysis (r = 0.92, p < 0.01). In contrast, the *Time-scaled Ramps* analysis resulted in CVR values that were anticorrelated with the end-tidal CO_2_ data, although this is not significant (r = − 0.75, p = 0.08). As by design, the *End-tidal CO_2_* analysis was not significantly correlated with the CO_2_ data (r = 0.51, p = 0.30). Irrespective of breath-hold model, the ICC values were higher in the data with greater variability in the breath-hold performance.

**Fig. 5:**
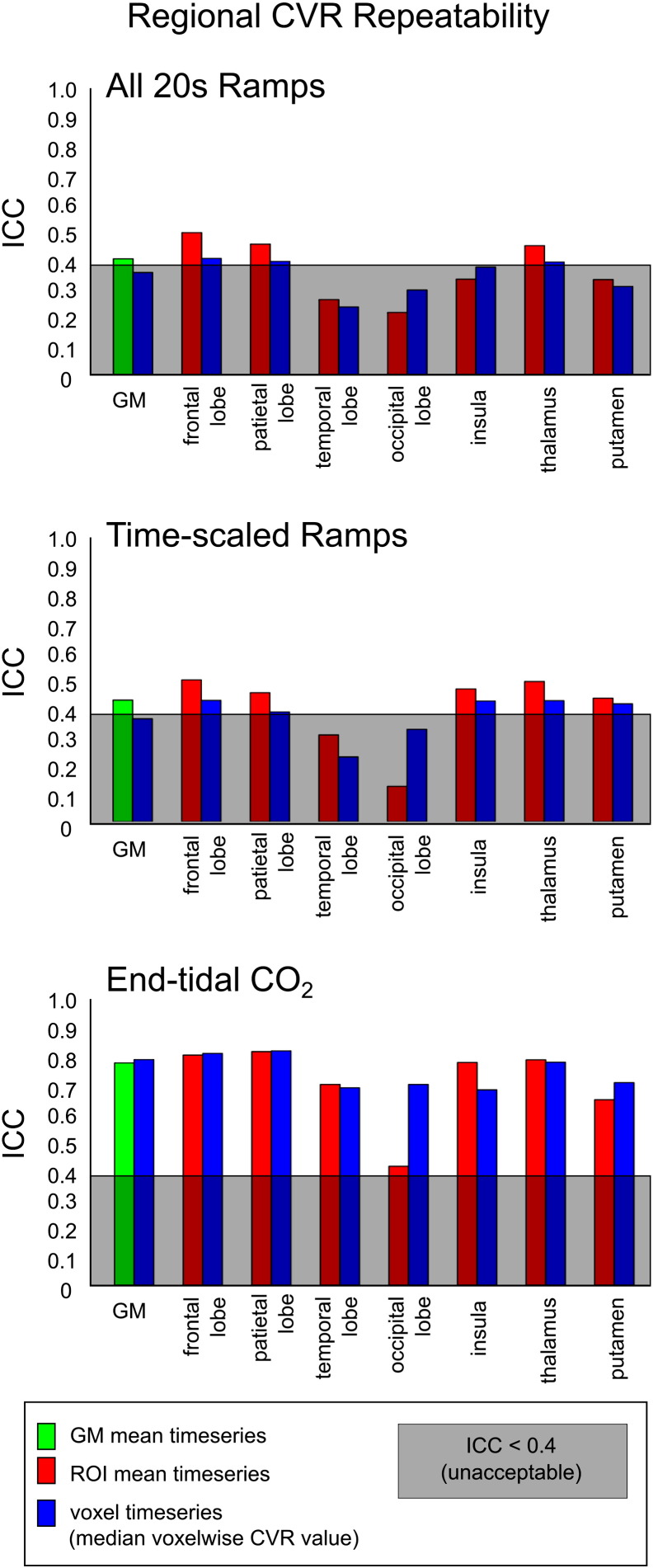
Repeatability of CVR values obtained for a region of interest. CVR was calculated within grey matter (GM) voxels and within 7 additional regions of interest. Values were calculated using both the GM or ROI mean timeseries and by calculating the median voxelwise CVR value. The ICC across the six breath-hold scans was calculated for both sets of results. ICC values below 0.4 (shaded grey) are considered to show “unacceptable” repeatability for fMRI experiments. Across the 8 regions considered, the *End-tidal CO_2_* analysis resulted in significantly greater ICC values, using ROI or voxelwise CVR values, compared to the *All 20s Ramps* and *Time-scaled Ramps* analyses (p < 0.0001, paired t-tests, Bonferroni corrected for multiple comparisons).

The ICC values obtained from the mean timeseries (GM and ROIs) and using the median voxelwise CVR value were compared (Fig. 5). There was no significant difference between the ICC values of the mean timeseries and voxelwise methods. The use of end-tidal CO_2_ regressors to calculate CVR resulted in higher ICC values than the *All 20s Ramps* or *Time-scaled Ramps* analyses in all regions tested, for both the mean timeseries and median voxelwise results (paired t-tests, all tests p < 0.0001, Bonferroni corrected for multiple comparisons). The ICC results for the insula, thalamus, and putamen ROIs are similar to results in larger ROIs (e.g., parietal, temporal lobes), suggesting that the size of ROI does not dramatically limit the robustness of the CVR measurement in this study.

### Spatial repeatability

**Fig. 6:**
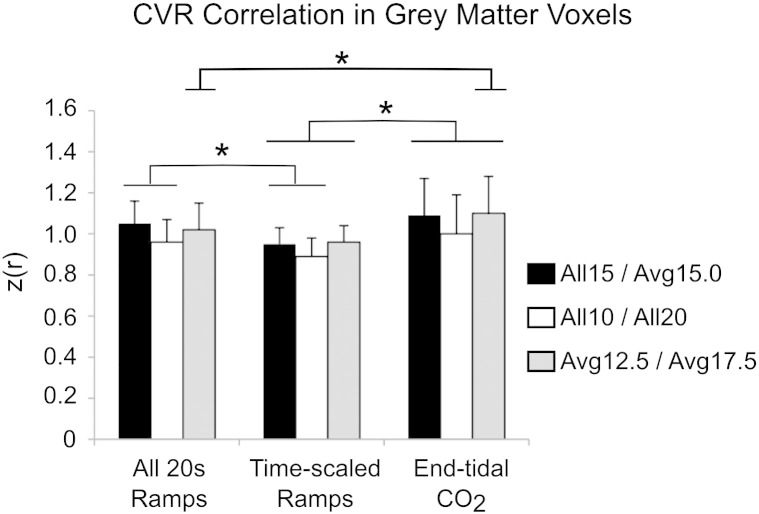
Group results of correlation analysis between pairs of breath-holding datasets that determine the effects of impaired consistency (*All15*/*Avg15.0*), impaired ability (*All10*/*All20*), or both (*Avg12.5*/*Avg17.5*). Voxels within GM showed greater z(r) correlation using the *End-tidal CO_2_* regressors than using the *Time-scaled Ramps* regressors in all comparisons. The *All 20s Ramps* model resulted in greater correlation than the *Time-scaled Ramps* model in the *All15*/*Avg15.0* and *All10*/*All20* comparisons. The *End-tidal CO_2_* model showed greater correlation that the *All 20s Ramps* model in only the *Avg12.5*/*Avg17.5* comparison (*p < 0.05, paired t-tests, corrected for multiple comparisons).

**Fig. 7:**
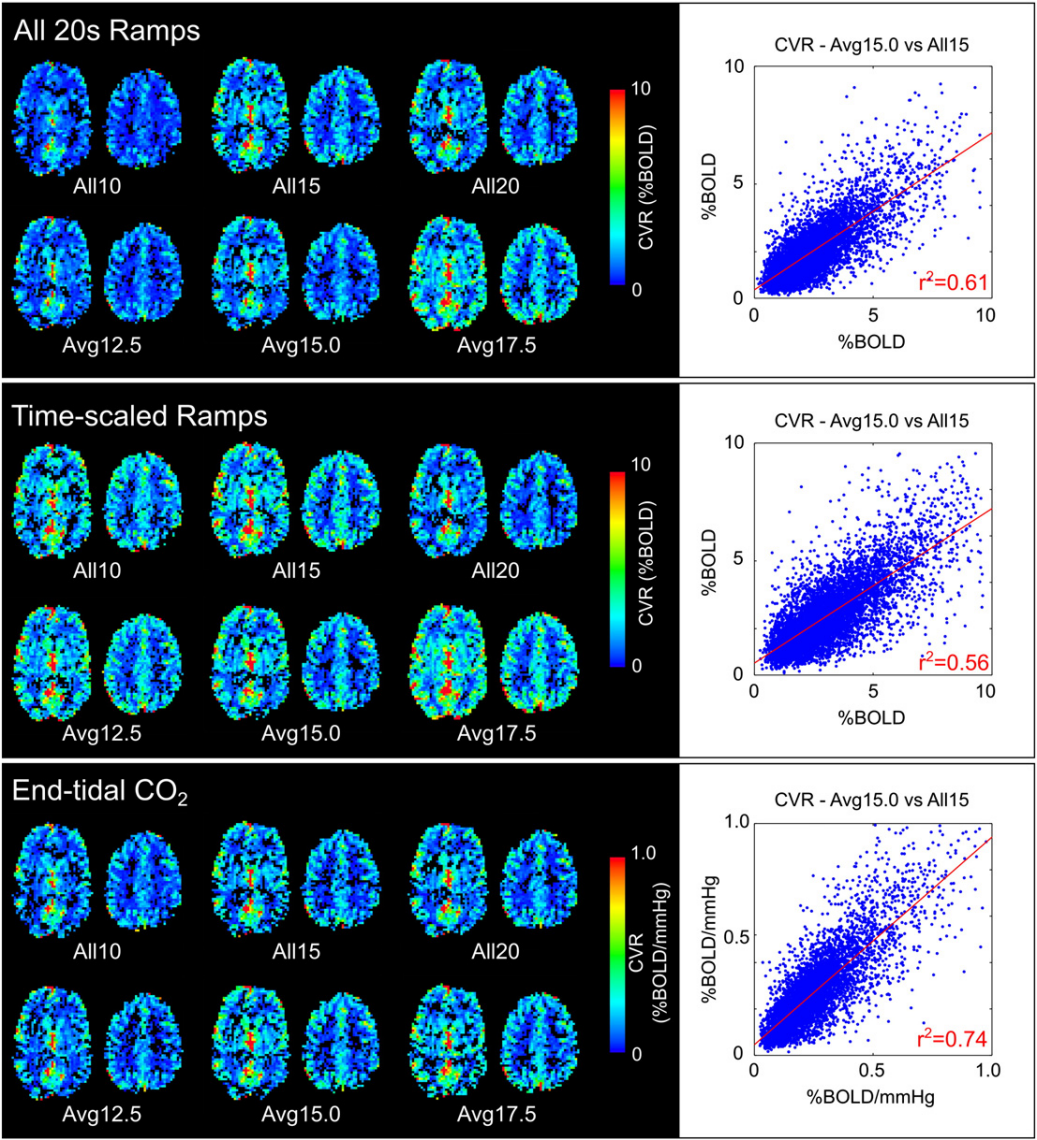
Example CVR maps for one subject. The *All 20s Ramps* and *Time-scaled Ramps* analysis result in CVR values with arbitrary units, whereas the *End-tidal CO_2_* analysis results in quantitative CVR values in units of %BOLD/mm Hg. All values within the modified grey matter mask are plotted for the *All15* and *Avg15.0* data, and the linear fit (red line) and correlation values indicated.

The group results of the spatial correlation analysis between pairs of CVR maps are shown in Fig. 6. The *End-tidal CO_2_* analysis resulted in significantly higher z(r) scores than the *Time-scaled Ramps* analysis in all three comparisons (the *Avg12.5*/*Avg17.5* comparison was also significantly more correlated following *End-tidal CO_2_* analysis than the *All 20s Ramps* analysis). The *Time-scaled Ramps* analysis resulted in significantly lower z(r) scores than the *All 20s Ramps* analysis in two of three comparisons. Examples of the CVR maps for one subject (identified as having the median correlation values in the *All15*/*Avg15.0* comparisons) are provided in Fig. 7. The ICC_spatial_ was calculated for each subject to test the reproducibility of CVR heterogeneity across all six breath-hold scans: the results are presented in Table 1. ICC_spatial_ was significantly larger in the *End-tidal CO_2_* analysis compared to the *All 20s Ramps* model (p = 1 × 10^− 5^) and *Time-scaled Ramps* model (p = 4 × 10^− 5^) (paired t-tests). Combined, these results show that the *End-tidal CO_2_* breath-hold model results in greater (and often significantly greater) repeatability of the voxelwise distribution of CVR values across grey matter.

## Discussion

### ICC and breath-hold modelling

We have simulated patient data (mimicking poor breath-hold performance) in healthy volunteers to show that BOLD CVR can be robustly mapped using breath-hold challenges, despite variation in challenge ability and consistency. All breath-hold regressor types assessed in this study resulted in significant ICC values (F-test, p < 0.05) in analysis of the GM timeseries. However, the results shown in Fig. 4 and the associated correlation analysis suggest that there are systematic confounds when using *All 20s Ramps*, and potentially the *Time-scaled Ramps*, to model the breath-hold response. The *All 20s Ramps* analysis resulted in CVR values correlated to the measured range in end-tidal CO_2_ of each scan, indicating these regressors do not account for variation in breath-hold ability (e.g., *All10* or *Avg12.5* data). This may lead to “false-positives” in patient studies, in which abnormally low CVR values may be measured due to difficulties performing the breath-hold task. In contrast, the results of the *Time-scaled Ramps* analysis show a trend towards anticorrelation with the end-tidal CO_2_ range. The CVR values obtained using *End-tidal CO_2_* regressors are, by design, the least correlated with the end-tidal CO_2_ variance (and challenge ability) across the six scans.

There is discrepancy in the literature over what constitutes an acceptable ICC for fMRI data. Although all of our ICC values reached significance (using CVR from the GM average timeseries), *the End-tidal CO_2_* regressors resulted in almost double the ICC of the other analysis techniques (0.82 versus 0.43 and 0.45). Existing literature provides numerous alternatives for reliability criteria in fMRI studies. Cicchetti and colleagues have proposed ICC groupings of poor (< 0.4), fair (0.41–0.59), good (0.60–0.74) or excellent (> 0.75) reliability (Cicchetti, 2001). This stratification has been adopted by more current fMRI research (van den Bulk et al., 2012), noting that these thresholds parallel the suggested acceptance levels of the neuroimaging community of ICC > 0.4 (Aron et al., 2006; Eaton et al., 2008). Under these guidelines, the *All 20s Ramps* and *Time-scaled Ramps* would provide “acceptable” but only “fair” CVR reliability, while the *End-tidal CO_2_* analysis provides “excellent” reliability.

When considering the three consistent (*All10*, *All15*, *All20*) and three varied (*Avg12.5*, *Avg15.0*, *Avg17.5*) scans independently, the ICC values for the GM timeseries were observed to be higher in the varied breath-hold duration scans. This finding suggests that it may be preferable to use breath-holding paradigms with varying challenge duration, even in healthy volunteers, in order to improve the robustness of the CVR measurement.

Finally, it is important to note that the *End-tidal CO_2_* model quantifies CVR measurements in units of %BOLD/mm Hg, rather than %BOLD. This normalisation enables better comparison between CVR values obtained in different sessions, or in different subjects, making cohort comparisons and longitudinal studies more robust.

### Regional CVR repeatability

The ROI analysis (Fig. 5) demonstrates that CVR can be reliably measured in smaller brain regions in addition to all of grey matter. The *End-tidal CO_2_* model continued to achieve acceptable ICC values (ranging from ICC = 0.45 in the occipital lobe mean timeseries to 0.86 in the parietal lobe using voxelwise analysis). The *All 20s Ramps* and *Time-scaled Ramps* results often slipped from “fair” to unacceptable ICC levels (ICC < 0.4). We conclude that regional CVR values require the *End-tidal CO_2_* model to achieve acceptable CVR repeatability.

To further understand the robustness of small scale CVR measurements within an individual subject, we assessed the voxelwise correlation between pairs of CVR maps and measured ICC_spatial_ values across all six CVR maps for each subject. The correlation analysis results (shown in Fig. 6) indicate that the results of *End-tidal CO_2_* analysis are significantly more repeatable between scans of different ability/consistency than the *Time-scaled Ramps* results, and some of the *All 20s Ramps* results. The ICC_spatial_ analysis (Table 1) also demonstrates that the *End-tidal CO_2_* model provides the most repeatable mapping of CVR variation within the brain.

### CVR and breath-holding in clinical applications

As described earlier, a meta-analysis of CVR literature has demonstrated a strong association between impaired CVR and stroke risk (Gupta et al., 2012). Silvestrini et al. (2000) provide one example of this connection, measuring correlation between CVR impairment and the level of risk that a patient with severe stenosis will go on to experience an ischaemic event. As surgical interventions to open stenosed or occluded vessels carry their own inherent risk of ischaemic events due to the release of microemboli, CVR could help stratify patients and quantify the relative risk and benefits of surgery of each individual. In a prospective study, Markus and Cullinane (2001) showed that severely reduced cerebrovascular reactivity predicted the risk of ipsilateral stroke and transient ischaemic attack (TIA) in patients with carotid occlusion (and to a lesser extent in asymptomatic carotid stenosis). These two examples used transcranial Doppler ultrasonography to measure CVR, whereas whole-brain BOLD fMRI can better localise the downstream effects of arterial stenosis and provide even more powerful diagnostic information. For example, CO_2_ gas administration and BOLD CVR maps determine the location and extent of abnormal vascular reactivity secondary to proximal large-vessel stenoses, including steal phenomenon, in moyamoya patients (Heyn et al., 2010; Mikulis et al., 2005). Breath-holds have also been successfully used to measure BOLD CVR in patients with brain tumours; good agreement with the results of gadolinium contrast administration was observed, and the authors suggest that BOLD CVR may be more sensitive to certain types of vascular abnormalities than the DSC technique (Pillai and Zacá, 2011, 2012).

With these compelling arguments for acquiring BOLD CVR maps in a range of patient groups, why is it not routine clinical practice? The difficulty in administering CO_2_ challenges and concerns regarding the repeatability of CO_2_ paradigms may be responsible for the limited implementation in clinical scenarios. Breath-hold paradigms have been administered successfully to paediatric patients with moyamoya disease (Thomas et al., 2013), suggesting that even patient groups that are notoriously difficult to scan can successfully complete breath-hold challenges in the MR scanning environment. The results presented in the current study indicate that poor performance of breath-hold challenges is not necessarily a limiting factor in robust mapping of CVR in patients; the *End-tidal CO_2_* analysis method compensates for ability and consistency factors and provides repeatable CVR values despite these performance variations.

### Considerations

Although the proposed breath-hold paradigm is relatively simple compared to breathing circuits and gas delivery systems, it may still cause problems for certain patient groups. For example, the use of a nasal cannula to record end-tidal values would be of little use if a subject did not (or could not) breathe through their nose. Small modifications to the experimental design (e.g., attaching the gas sampling line to a face mask with minimal breathing resistance) could extend the suitability of the breath-hold challenge without greatly impacting the ease of use. However, there will always be patient groups that lack the minimal compliance needed for a breath-hold challenge (e.g., patients with dementia), and gas inhalation techniques may be the only option for these individuals.

In addition, it is important to acknowledge that all respiratory challenges can simultaneously alter oxygen levels in addition to CO_2_ levels: a breath-hold challenge results in mild hypoxia as well as the intended hypercapnia. Although oxygen is vasoactive, there is minimal impact on cerebral blood flow within an end-tidal O_2_ range of 60–150 mm Hg (Brugniaux et al., 2007). However, changes in arterial oxygen can directly influence the BOLD contrast (Bulte et al., 2007). To assess how oxygen is altered in our data, we assessed the baseline values and ranges of end-tidal O_2_ data (results presented in the Supplemental Figure), and compared the end-tidal O_2_ timeseries with the end-tidal CO_2_ timeseries in each scan. The O_2_ and CO_2_ data were significantly negatively correlated (r values ranging from − 0.44 to − 0.98; p < 0.1 × 10^− 4^; corrected for multiple comparisons). Thus, the effects of hypercapnia and hypoxia cannot be disentangled in our data. The extent of hypoxia associated with the breath-hold challenges (indicated by the range) is enhanced with breath-hold duration, and could potentially cause an artifactual enhancement of BOLD CVR values. A recent study by Tancredi and Hoge (2013), utilising a nearly identical breath-hold paradigm, observed that the consequent mild hypoxia did not significantly influence the CVR results relative to gas inhalation methods that did not have a hypoxic component. We therefore assert that the hypoxic component of breath-hold challenges is not significant in the current study. We encourage future studies to monitor both end-tidal O_2_ and end-tidal CO_2_ in order to determine whether transient hypoxia becomes critical in a given patient group.

Finally, a recent study has demonstrated that the reactivity response to CO_2_ is sigmoidal, and the baseline CO_2_ level determines whether the reactivity response is in a linear or non-linear domain (Tancredi and Hoge, 2013). As demonstrated in that study, and in Fig. 3, the paced breathing during the breath-hold scans resulted in mild hypocapnia compared to resting levels. This shift in baseline may thus impact our reactivity measure. It is unlikely that this is a major factor in our data. First, due to differences in paced breathing rates between the published study and our own paradigm, we observe only − 5 mm Hg changes in baseline CO_2_ levels compared with − 10 mm Hg hypocapnia observed in the Tancredi and Hoge paper. Secondly, we have limited our CVR analysis to relative changes in %BOLD signal; Tancredi and Hoge observed significant effects of baseline CO_2_ on absolute reactivity measures but these were negligible when considering relative measures. Finally, if the shift in baseline physiology moved us into the non-linear regime of a subject's dose–response curve, than we would expect that end-tidal CO_2_ regressors describing breath-hold challenges of varied effect sizes would no longer fit the data robustly, reducing repeatability in group ICC analysis. The excellent ICC values achieved using the *End-tidal CO_2_* analysis method indicates that the influence of a baseline shift in CO_2_ levels is not significant in our study. However, this may no longer hold true in patient groups with altered physiology (Bright et al., 2010; Zhao et al., 2009). We recommend that the paced breathing rate be targeted to match a patient's natural breathing rate as closely as possible, to minimise the baseline hypocapnic effect.

## Conclusions

This study demonstrates that BOLD CVR can be mapped using breath-holds, with high inter- and intra-subject repeatability, despite variations in the duration and consistency of breath-hold performance. High repeatability is achieved by incorporating end-tidal CO_2_ data into the CVR analysis. Because end-tidal CO_2_ data represent the physiological effects of the breath-hold challenge, regardless of how the challenge was performed, any patient “misbehaviour” can be accounted for. We offer the following guidelines for future work incorporating breath-holds into studies of difficult patient cohorts:

• End-tidal CO_2_ data *must* be acquired and incorporated into CVR analysis to account for variations in breath-hold performance (and inter-subject differences in physiology) to achieve repeatable measures across the brain.
• Patients can end breath-holds prematurely if needed; however, it is important to stress that they briefly exhale before resuming normal breathing in order to obtain the most accurate end-tidal CO_2_ values possible.
• An ideal breath-hold stimulus would involve end-expiratory challenges interleaved with periods of paced breathing. Details of the paradigm can be adjusted to suit the target patient cohort (e.g. the paced breathing could be manipulated to match the resting respiratory rate of the subject, or auditory instructions could be supplied to subjects with poor vision).

By incorporating end-tidal CO_2_ data into the acquisition and analysis procedures, breath-hold challenges achieve robust CVR maps even in “difficult” subjects. These results support the use of breath-holding for measuring CVR in clinical studies, and will hopefully encourage more widespread implementation of this important diagnostic test.

The following are the supplementary data related to this article.

**Supplementary Figure:**
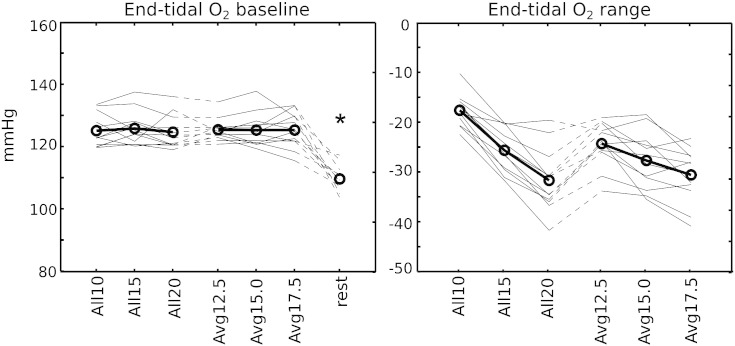
Summary of the end-tidal O_2_ effects associated with the six breath-hold scans. The baseline and range of end-tidal O_2_ values for each subject are shown and the group mean plotted (circles). The baseline end-tidal O_2_ values were significantly higher in all six breath-hold scans compared to the resting scan (*p < 0.0005, paired t-tests, Bonferroni corrected for multiple comparisons).

## Figures and Tables

**Table 1:** Effect of CVR analysis method on the repeatability of the spatial distribution of voxelwise CVR values. Using the voxelwise CVR maps, ICC_spatial_ was calculated for each subject and transformed into a z(r) statistic. The *End-tidal CO_2_* analysis resulted in significantly greater z(r) values across the population relative to the *All 20s Ramps* (p = 1 × 10^− 5^) and the *Time-scaled Ramps* (p = 4 × 10^− 5^) analyses.

Repeatability of CVR spatial distribution (ICC_spatial_)			
Subject	*All 20s Ramps*	*Time-scaled Ramps*	*End-tidal CO_2_*
1	0.87	1.10	1.13
2	0.69	0.63	1.02
3	0.95	1.02	1.26
4	0.73	0.60	0.87
5	0.66	0.78	1.00
6	0.78	0.85	1.02
7	0.91	0.93	1.07
8	0.71	0.78	0.89
9	0.78	0.55	0.93
10	0.79	0.71	0.97
11	1.00	0.60	1.07
12	1.05	0.66	1.10
mean	0.82 ± 0.13	0.77 ± 0.18	1.03 ± 0.11
